# Nourishing food, clean air and exercise: medical debates over environment and polar hygiene on Robert Falcon Scott’s British National Antarctic expedition, 1901–1904

**DOI:** 10.1017/mdh.2024.3

**Published:** 2024-07

**Authors:** Edward Armston-Sheret

**Affiliations:** Institute of Historical Research, School of Advanced Studies, University of London, Senate House Malet Street, London, WC1E 7HU United Kingdom

**Keywords:** Hygiene, exploration, Edwardian, polar, Antarctic, scurvy

## Abstract

The late nineteenth and early twentieth century saw dramatic new developments in climatic medicine, particularly the institutionalisation of thinking about tropical hygiene. There were also more limited efforts to understand how hygiene theories should be applied in a polar environment. Studying the British National Antarctic Expedition (1901–1904), led by Robert Falcon Scott, helps us understand how these practices had both similarities and differences from applications of hygiene in other contexts. The expedition offers unique insights into debates about hygiene, environment, and health because of the important, and well documented, role that medics, naval officers and scientists played in organising logistical arrangements for the journey to Antarctica. In analysing the writings of expedition members and organisers, this paper examines the ways that the universal tools of hygiene theories were applied and developed in a polar environment. Many of the most acute threats seemed to come not from the outside environment but from the explorers’ supplies and equipment. There was general agreement on many issues. Yet the expedition’s organisers, medics and leadership had numerous arguments about the best way to preserve or restore health. These disagreements were the product of both competing medical theories about the cause of disease and the importance of embodied (and somewhat subjective) observations in establishing the safety of foods, atmospheres and environments in this period.

The British National Antarctic Expedition (BNAE, 1901–1904) is often heralded as the starting point of the ‘heroic age’ of polar exploration. When the expedition’s ship, *Discovery*, left New Zealand in 1901, on board were individuals who would go on to be household names – including its leader Robert Falcon Scott and its third Lieutenant Ernest Shackleton. Unsurprisingly, most writing about the expedition focuses on the biographies of these individuals and the adventure of the ‘race’ to the South Pole. But this paper argues that the expedition can be used to tell a different story, which allows us to understand how medics, scientists and explorers sought to systematically apply hygiene theories in a polar context. I demonstrate that such regimes of hygiene focused on food, air and exercise. This paper is also concerned with the relationship between medical theory and practice. I show that while there was a consensus on many key pillars of expeditionary hygiene, there were significant disagreements on how to apply these theories and assess risks in practice. In large part, this was because much of the important work of expeditionary medicine relied on subjective sensory assessments and personal embodied experience.

Much writing about exploration has focused on the biographies of famous individuals. There is, however, a vibrant and growing subfield of literature that has sought to use exploration to understand changing approaches to medicine and health. Scholars, most notably Vanessa Heggie, have examined the relationship between expeditions and the development of extreme environment physiology.^1^ Others have focused their attention on how expeditions shaped understandings of nutrition and diet.^2^ As such literatures show, expeditions are both illustrative of and played a key role within broader medical developments in the nineteenth and early twentieth centuries.

I argue that focusing on the BNAE can help us to develop new insights about the relationship between hygiene and health at the turn of the twentieth century. The BNAE travelled to Antarctica on *Discovery*, a specially built ship, arriving in early 1902 and departing in early 1904.^3^ This vessel was manned by a slightly varying crew of just under forty officers, scientists and sailors. Once in Antarctica, the expedition overwintered on board the ship and carried out a programme of geographical exploration and scientific research. They conducted several significant overland journeys, such as the first trip to the Polar Plateau. Scott, Shackleton and the expedition’s junior medic, Edward Wilson also completed a journey over the Ross Ice Shelf that set a new farthest south record, coming closer to the South Pole than any previous humans.

The BNAE is a particularly good case study through which to understand Edwardian approaches to polar hygiene, as it was jointly organised by the Royal Society (RS) and the Royal Geographical Society (RGS). Because of this institutional involvement, we have unprecedented knowledge about how it sought to care for the explorers’ bodies. The societies established committees to oversee the organisation of the expedition, which led to more meticulous and bureaucratic record keeping than any comparable private venture.^4^ As we shall see, the committees also drew on the expertise of other travellers and medics, meaning the expedition can offer insights into broader medical thinking in this period. The BNAE also influenced British cultures of polar exploration: Shackleton led three further polar expeditions, while Scott led one final (and for him fatal) journey to Antarctica, employing many of the BNAE’s crew and scientific staff on it. This expedition shaped those that followed it.

I begin to examine this subject by surveying previous scholarship on hygiene and expeditionary medicine. I then move on to examine the BNAE and the most important aspects of expeditionary hygiene: food, air and exercise.

## Hygiene and environment

One of the most important theories that shaped the expedition’s approach to health was the idea of hygiene. This topic was considered so essential that the RGS and RS organised a specific ‘hygiene committee’ to oversee the expedition’s approach to this subject. The first two meetings of the committee were chaired by the pioneer of antiseptic surgery and president of the RS, Lord Joseph Lister.^5^ By 1899, president of the RGS, Sir Clements Markham, described the committee arrangement as ‘a very ponderous machine’. ^6^ As the window for the expedition’s departure approached, the delays produced by this machine became untenable. In early 1901, Scott applied to take control of the provisioning of the expedition, and the committees were disbanded.^7^ From this point on, Scott was responsible for logistical decisions, although he had long been delegating many aspects to the expedition’s surgeon and bacteriologist, Dr. Reginald Koettlitz.^8^ Once in Antarctica, much of the work of keeping the expedition healthy was carried out by the expedition’s two medics, under the overall leadership of Scott. But even after the disbandment of the formal committee, hygiene remained the primary method used to preserve good health on board the ship.

What did hygiene mean in this period? Dr. George Wilson neatly summarised the logic behind such ideas in his (1873) manual, *A Handbook of Hygiene.* He argued that hygiene meant ‘the avoidance of disease by the removal of its avoidable causes’. Clean living had both moral and medical dimensions. The social causes of disease, according to Wilson, were multifaceted, including ‘intemperance, immorality, injudicious marriages and excesses of every description’. On the medical side, Wilson was concerned with specific vectors of disease, such as ‘impure air, impure water’ and ‘unwholesome food’ but also with environmental conditions such as ‘dampness of soil, deficiency of warmth, etc.’^9^ Because of their concern with cleanliness, such theories were compatible with the newly emerging ‘germ theories’ of disease causation, which assumed greater prominence in the later part of the nineteenth century. But they were also compatible with older miasmic theories of illness that attributed ill health to impure air.^10^ Over the course of the nineteenth century, hygiene theories and new sanitation technologies were applied in (and adapted to) various settings and at different scales. These applications ranged from domestic regimes of cleaning and citywide public health campaigns to commodities and ideas that circulated through global networks.^11^ For instance, both the Royal Navy and European explorers developed specific approaches to hygiene.

Naval thinking about hygiene had a marked influence on the BNAE. Not only was the expedition organised along naval lines, but both Scott and most of the *Discovery*’s crew were drawn from this institution. The historian Elise Judza Smith has studied how the move from wind to steam power shaped the development of the Royal Navy’s practices of hygiene between 1840 and 1900.^12^ Smith argues that ‘preventative medicine at sea revolved around maintaining a clean, dry and well-ventilated environment in spaces that were invariably overcrowded, damp and enclosed’. The ship, then, presented particular challenges for hygienic practice, as it trapped in the ‘noxious emanations from decomposing animal or vegetable matter’ that were considered responsible for causing outbreaks of disease.^13^

Through the nineteenth century, the growing influence of microbiological theories of illness shifted attention away from the threats of miasmic gasses, but also ensured that ideas of hygiene and cleanliness remained important.^14^ In theory, Smith argues, the ‘sealed’ environment of the ship made hygiene an attractive solution to illness and disease: if hygienic standards could be maintained, good health could be ensured even on long voyages.^15^ In practice, she notes that naval surgeons reported outbreaks of disease even on clean ships. There were also concerns about the effects of tropical environments on sailors’ health.^16^ Below, I develop on this work by examining issues of health and hygiene in a different environmental context and with greater attention to questions of food and provisioning.

Approaches to hygiene were shaped by ideas about empire, race and environment.^17^ The ‘tropics’ were seen as areas where European travellers had to be particularly careful.^18^ The historical geographer David Livingstone has demonstrated that in the late nineteenth century hygiene theories were applied to try to enable European bodies to maintain their vitality while travelling in and colonising the tropics.^19^ Hygiene encompassed a set of practices that would help to protect European bodies from the perceived negative effects of close and prolonged contact with warm climates and their inhabitants.^20^ Europeans could, the argument went, live in the tropics successfully only if they adopted rigorous regimes of hygiene to prevent contamination by tropical diseases as well as moral regulations to prevent close contact with local people.^21^ Regimes of hygiene in such locations addressed travellers sexual behaviours and their drinking habits.^22^ From this perspective, hygiene was reasoned as a modus operandi for travellers, explorers and colonial officials to limit and restrict their contact with the environments through which they travelled. Interestingly, these ideas became increasingly formalised around the time the BNAE was being organised. The London School of Tropical Medicine was founded in 1899.^23^ While these broader approaches to hygiene and climatic medicine shaped the organisation of the expedition, it was also influenced by the experiences of previous polar travellers.

In a polar context, one of the primary concerns was scurvy. This focus is interesting, as this was far from the only threat the explorers would face when they reached Antarctica. They would need to grapple with a variety of issues including frostbite, snow blindness and hypothermia. But these other afflictions received surprisingly little attention in the BNAE’s discussions about hygiene, particularly before the departure of the expedition. Why was so much attention devoted to scurvy?

Part of the answer lies in scurvy’s unpleasant symptoms and the cultural meanings attached to these. As Jonathan Lamb has argued, scurvy was a shameful affliction, associated with dirt and depression.^24^ The disease had a range of physical and mental effects that made it a particularly terrifying condition to suffer from on a polar voyage, including depression, madness, open sores, blackened limbs, ‘foul ulcers’, receding gums, loose teeth and, if untreated, death.^25^ The disease also produced unpleasant mental symptoms evocatively described by the naval physician Thomas Trotter: ‘The mind in the beginning of the disease is timid and desponding, but towards the end of the fatal period there is total indifference and seeming torpor of every feeling’.^26^ Suffering from scurvy resulted in the physical and mental disintegration of the body in terms very different from how heroic explorers wished to be understood.

The other reason that scurvy attracted more attention than other issues was that its causes were heavily debated at the time. We now understand that scurvy is caused by a shortage of vitamin C. However, this was not understood in the nineteenth century. In the late eighteenth century, the Scottish naval medic James Lind had conducted experiments that showed the effects of citrus fruits (which we now know are high in vitamin C) in preventing and curing the disease.^27^ This breakthrough led to the use of lemon and lime juice within the Royal Navy in the late eighteen century, which dramatically reduced cases of scurvy.^28^ But, as vitamins were not discovered until the 1930s, there was little understanding of why citrus fruits worked. Belief in the efficacy of lime juice declined over the course of the century, as practices of storage and preservation (such as boiling) often destroyed much of the vitamin C contained in it.^29^

Outbreaks of scurvy on board ships with a lime juice ration led to efforts to find an alternative explanation for the disease, leading to suggestions it was caused by impure air or poor diet.^30^ The shortening of many maritime journeys due to the emergence of steam power and general improvements in diet complicated efforts to prove lime juice’s efficacy.^31^ By the latter half of the nineteenth century (and even into the early twentieth century), maritime scurvy was far less common than it had been in earlier periods, but its exact causes were poorly understood.

Hygiene was seen as the most important means to prevent and treat this affliction on polar expeditions. In 1858, the Irish naval officer Alexander Armstrong published *Observations on Naval Hygiene and Scurvy: More Particularly as the Latter Appeared during a Polar Voyage.* The book was based on his own experiences in the Arctic, searching for the lost explorer Sir John Franklin in the early 1850s. As the title of this work implies, he understood hygiene (covering diet, exercise and ventilation) as the way to prevent ill health on polar expeditions. Armstrong’s ideas reflect and shaped more general naval thinking about hygiene and scurvy: he highlighted the importance of lime juice in preventing it as well as the necessity of an ample and varied diet. But Armstrong suggested that the disease could be made worse by environmental factors, particularly ‘impure air’, which he argued ‘always tends to impart a more aggravated character to Scurvy’.^32^ He argued that exposure to damp, moist air often caused outbreaks of the disease alongside other conditions, particularly rheumatism. He also highlighted the importance of moderate exercise in preserving good health.^33^ As I now examine, these broad ideas influenced attitudes towards hygiene on the BNAE, but also produced vigorous debates.

## Nourishing foods

‘Give the men good healthy food and I guarantee you will have no diseases’, the Norwegian Arctic explorer Fridtjof Nansen wrote to Koettlitz in late February 1900.^34^ Koettlitz was working with Clements Markham and his cousin Admiral Albert Markham on a report to be presented to the hygiene subcommittee in May 1900, giving their recommendations on the BNAE’s food supplies and other matters of hygiene.^35^ When completed, the report proposed a diet consisting of ‘bread and biscuit, salt beef one day a week, salt pork two days a week, and preserved meats for four days a week, to which peas[,] vegetables, soups, and fruits can be added’.^36^ These preserved supplies, the report suggests, could be supplemented with fresh live meat from New Zealand and, possibly, ‘seal and penguin flesh sufficient to last through the winter’.^37^ The prospect of eating seal and penguin meat is not discussed further by the subcommittee, suggesting there was little concern about eating locally hunted food.

The report to the hygiene subcommittee also dealt with topics that are well beyond the realms of twenty-first century thinking about hygiene – reflecting the moral dimensions of hygiene theories in this period. They were concerned about both the taste and variety of the food supplies and drew on their personal experiences to support their claims about the relative benefits of different items. For instance, in the report, Clements Markham notes that preserved potatoes are ‘generally not good’ but that frozen potatoes were reported to be ‘perfectly good as regards to taste’ if prepared correctly.^38^ Markham also claims that ‘[s]ome kinds of food lose flavour in low temperatures, others do not’, suggesting that ‘[t]ests to ascertain this can be applied’.^39^ Indeed, Markham’s stressed the importance of variety, emphasising that the ‘preservation of health’ on previous expeditions was ‘attributed almost as much to the variety, as well as to the excellence of provisions’.^40^ Markham clearly considered having a varied and flavoursome diet central to the health of the expedition, suggesting that, like tropical hygiene, regimes of polar hygiene were linked to issues of morale and mental health

Koettlitz shared the idea that variety was essential. He had travelled in the Arctic as part of the Jackson–Harmsworth expedition (1894–1897) and drew on his personal experiences even more extensively. He wrote to Scott claiming that the ‘qualities and variety’ given in a later draft of the provisions list reflect his ‘recollections, only too vivid, as to the vagaries and capriciousness of the appetite during the Arctic Winter’.^41^ For Koettlitz, the polar winter posed a threat to the explorer, undermining their ability to control their appetite, a loss of control that would have both physical and psychological consequences. He goes on to stress the importance of a large supply of ‘vegetables, fruit, preserves, and sugar’ claiming that sweet foods are vital for maintaining both ‘bodily heat’ and ‘muscular strength’.^42^ He also saw food as psychologically important and notes that ‘the appetite for these so frequently becomes a craving which is difficult to satisfy unless there is an abundance of these actual necessities in a polar climate’.^43^ In his eyes, polar life could have disturbing and unpredictable effects upon the explorers’ relationship to food, and without varied supplies it may not be possible to satisfy these tormenting cravings. What is interesting about his approach is the importance he attaches to his personal experience of cravings (rather than scientific tests or theories about diet) in his assessments about what sort of foods to take. Hygiene was about trusting your gut.

Scott’s views on food are harder to pin down. In his published account, he noted that he disagreed with Nansen (and implicitly Koettlitz) on the importance of a varied diet. He claimed that on sledging journeys ‘the food was pretty much the same day after day, and though we sighed for changing quantity we never particularly desirous of changing the quality’.^44^ While Scott’s comments here refer to sledging foods, there are other suggestions that his and Koettlitz’s views of food differed. Koettlitz, in his November 1900 letter to Scott, writes that ‘having plenty of varied as well as wholesome food’ was one of the best means of preserving health. He therefore criticised Scott’s efforts to cut costs and variety in the expedition’s provisions, claiming they were ‘penny wise and pound foolish’.^45^ At other points, Scott seems to have been more attuned to the psychological role of food in the preservation of expeditionary morale, such as through the use of special and varied foods in mid-winter and Christmas celebrations.^46^ While Koettlitz, and Markham were clearly advocates of the physiological and psychological importance of a varied diet, Scott’s views appear more ambiguous.

When it came to the physical properties of the expedition’s food supplies, the similarity between the dietary proposed in Markham’s report and previous nineteenth-century polar expeditions caused anxiety.^47^ Many expeditions adopting a similar diet had experienced problems with their food supplies, including outbreaks of scurvy. These concerns were directed at one food in particular: preserved meats. Salt meat was, after advice from Nansen and Lister, ruled too dangerous to take altogether, while tinned food was considered a risky but essential item.^48^ Lister’s views on this subject may have been influenced by the fact that he had recently read a paper before the Royal Society by Frederick G. Jackson (leader of the Jackson–Harmsworth expedition), which argued that poorly preserved meat caused scurvy.^49^ These concerns perhaps also reflected growing anxieties about food poisoning in Britain at this time.^50^

The ‘danger of tinned food’, according to Markham, lay in its potential to cause both ‘ptomaine and metallic poisoning’. ^51^ Ptomaine, a substance believed to be produced by decaying meat, was held by some to cause scurvy, while metallic poisoning may have led to psychological and physical breakdown on other polar expeditions. Preventing contamination from the expedition’s tinned food, therefore, became a key concern of the committee. To combat these perceived dangers, Markham argued that all tins and other food containers should be tested to ensure the ‘absence of lead in any from or other deleterious substance’ and suggested that ‘the purity of the food itself’ should also be checked.^52^ Indeed, Markham claimed that these tests were ‘absolutely necessary’. ^53^ The hygienic status of tinned food was far from assured because the explorers would have little knowledge about the conditions under which it had been produced and stored. While tinning allowed food to be consumed ‘out of season and out of place’, this was something that provoked anxiety rather than celebration amongst the expedition’s organisers.^54^ Reliance on tinned food raised questions about which technologies, companies and tests to trust.

The expedition’s organisers took concerns about the purity of tinned food seriously and carried out several tests to establish the safety of different supplies. This involved members of the hygiene committee personally consuming the potential provisions and consulting adverts and articles in medical journals such as *The Lancet.* ^55^ Neither of these tests was sufficient to assure a food’s hygienic status, and the subcommittee was asked ‘to suggest a suitable analyst’ to carry out scientific tests on the expeditions’ food supplies.^56^ Evaluating the hygienic status of food, and its potential effects upon the body, was both a serious matter and something that required scientific knowledge.

Although there was a general agreement that tests should be carried out to ensure the food taken was safe, deciding which foods should be tested and what sort of analysis should be applied was less straightforward. The hygiene subcommittee liaised with T. E. Longhurst at the Government Laboratories in the hope of getting his department to carry out tests. Longhurst felt that the chemical and microscopic examination of all the expedition’s food supplies was unnecessary. He suggested the chemical inspection of a limited – and seemingly random – selection of the expedition’s provisions, including pepper, baking powder, tea, rum and tinned milk.^57^ The rest of the tinned food, Longhurst argued, should be inspected by the Navy’s Victualling Yards.^58^ In the end, chemical tests were only carried out on tender samples of pemmican, preserved milk and other specialty foods.^59^ The results suggest that concerns about quality were justified: one sample of pemmican was found to contain ‘pieces of steel, probably from the machinery with which prepared, weighing rather more than half an ounce’.^60^ The other examinations carried out by the Government Laboratories did not address whether any microbes or parasites were present in the samples, but only assessed their fat, sugar and protein content.^61^ Equally, none of the food actually taken aboard the *Discovery* was looked at by Longhurst.^62^ The much-touted ‘scientific tests’ did little to establish whether or not the expedition’s food supplies were safe.

Instead of being tested by the Government Laboratory, the food to be taken on board the *Discovery* was examined by Mr. H. Spadaccini, an inspector at the Port of London Sanitary Authority. Through the months of June and July 1901, Spadaccini rushed around the expedition’s warehouse in the East India Docks checking every case of supplies.^63^ He inspected all tins externally and opened three tins from each case order to examine their contents for signs of decay. ^64^ These examinations relied not on scientific instruments but on his sensory observations of sight, smell and possibly also taste. Spadaccini’s letters to Scott give us a fascinating insight into the reasons for rejection, meticulously listing the number of ‘blown’, ‘collapsed’, ‘leaky’, ‘rusty’ and ‘doubtful’ tins in each case.^65^ The vast majority of the expedition’s tinned food was supplied by the large Scottish company Maconochie.

Problems soon began to emerge with the provisions they supplied. Of the 450 cases of tins provided by Maconochie, 116 cases were rejected at the first examination and 49 at the second examination. ^66^ In total, 165 cases, more than a third of the total supplied by the company, were rejected by Spadaccini.^67^ On one level, the rejection of these spoiled tins should have reassured Scott and the expedition’s medics about the quality of the tinned food taken on board. But Spadaccini’s last notes to Scott reveal the unstable nature of the provisions, a problem that would haunt the expedition. On the 19 July 1901, soon before the expedition was due to depart, Spadaccini wrote to Scott that he had an ‘uneasy feeling’ after finding a blown tin in some of the cases he inspected previously and found ‘apparently good’.^68^ The tinned food supplies were not a stable product that once inspected could be labelled as safe; they needed to be re-inspected at different points to check if they had deteriorated.

With the failure of the examinations in London to establish which foods could be considered hygienic, the testing of the food was deferred across time and space. Spadaccini advised Scott that a consignment of twenty-six ‘doubtful’ tins, which due to time constraints had to be taken on board the *Discovery,* should ‘be received subject to examination in Australia and payment on result’.^69^ This solution, Spadaccini suggests, would mean the tins would be subjected to ‘the extra test of a passage through the tropics’ before inspection.^70^ For Spadaccini, the tropics were a climatic test of purity, and if they could pass through this region without decomposing, then they would be safe to consume in Antarctica. This idea that the environment would test tinned food and reveal any underlying contamination was also held by Koettlitz. Later, in Antarctica, after further concerns about the quality of tinned food emerged, he wrote to Scott advising him that if he had to make a selection amongst the tinned food, then ‘preference might be given to those which have been twice through the tropics’, as this test would have helped to expose any underlying problems with them.^71^ The tropical climate, then, replaces both scientific testing and inspection in London as the method for establishing the hygienic status of food – its extremes of heat and humidity rendering visible any latent taint that would be impossible to establish in other environments.

Many tins spoiled on the journey to New Zealand, meaning a significant but unrecorded quantity of unsafe tins were left there.^72^ Even after these efforts, the explorers found that many tins were inedible. Scott notes that one of the ‘regular duties’ of the expedition’s two doctors was the examination of ‘[e]very tin of food … after it is opened and before it is served out’.^73^ This task was a sensory affair as much as a scientific one, relying on the doctors’ taste and smell. Wilson did not enjoy it, writing in his diary that it involved tasting ‘mouthful after mouthful of sour milk’, a job which required him to ‘have a bucket handy always’.^74^ In contrast to milk, tins of meat, soup or fish had to be inspected ‘nasally only’, rendering this a slightly easier duty.^75^ In the explorers’ eyes, establishing whether tinned food was safe to consume relied on a specialist, sensory and embodied medical knowledge. But the fact that this task was carried out by the doctors can also be read as demonstrating a lack of trust in the expedition’s cooks to detect and discard tainted food. Scott described the expedition’s second cook, Henry Brett, in dismissive terms claiming that he ‘is a thorough knave, but, what is even worse, he is dirty – an unforgivable crime in a cook’.^76^ Scott clearly placed little trust in Brett’s ability to cook hygienically. Again, we see the importance of relationships of trust in establishing what was safe to eat.

With much of the expedition’s food rendered suspect, it is small wonder that the explorers seized opportunities to cook and catch food themselves. Many officers found the small snacks of toasted bread ‘the most enjoyable meal of the day’.^77^ Equally, polar meats – seals and penguins – became an increasingly important part of the men’s diet, particularly after the discovery of scurvy cases in October and November 1902.^78^ Unlike the tinned foods, the explorers could ensure that such meats were prepared in a hygienic way. The explorers killed large quantities of seals and grew to prefer these polar meats to their tinned food supplies. Albert Armitage considered Weddell seals ‘real good eating, though I cannot say that I ever went so far as to declare it equal to fresh beef or mutton as some in our company did’.^79^ Concerns about the hygiene and safety of food supplies pushed the explorers to suspect industrially produced food and into closer contact with polar animals and the environment.

## Clean air and exercise

Another major concern about hygiene on the expedition was the ‘warmth, dryness, [and] ventilation’ of the crew’s living quarters on the *Discovery.* ^80^ Scott viewed ventilation as important, noting that it ‘must always be a subject of serious consideration to polar explorers’.^81^ The explorers’ concerns were based on the importance of air quality within medical thought during this period. A nineteenth-century naval textbook argued, for instance, that poor ventilation on board a ship led to the imperfect oxygenation of the crew’s blood and, more dangerously, ‘the absorption of effete matter from neighbouring bodies closely crowded together’.^82^ The body’s own waste products were considered particularly dangerous, and campaigns for better ventilation of buildings were a major theme in public health campaigns during this period. Peter Baldwin notes that those who slept in unventilated spaces were considered to be ‘slowly suffocating in a toxic fog of their own breath, sweat, and flatulence’.^83^ On a wooden ship like the *Discovery*, the ‘foul air’ and bad smells from decomposing matter in the bilges were a source of concern, requiring constant cleaning and pumping.^84^

In the eighteenth century, the Royal Navy had experimented with ventilation equipment on board its vessels, hoping these technologies would reduce the high mortality rates of its crews in the Caribbean. These technologies proved cumbersome and ineffective. Their purpose also changed. Initially, the purpose of these devices had been to increase the flow of outside air into the ship. But shifting ideas about the ability of Europeans to ‘acclimatize’ to tropical environments meant that from the early nineteenth century the goal of ‘ventilating machines became to ‘*insulate* British bodies from inhospitable [i.e., tropical] environments’.^85^ The efforts of the explorers to deploy these technologies illustrate how ventilation was viewed quite differently on board a ship in the polar regions. Here, outside air was often viewed as healthy and restorative.

The problem lay in the fact that a ‘cold, damp’ draught was considered by many medical experts to ‘be even more injurious than stale air’, particularly if blowing on a person while they were sleeping.^86^ Naval textbooks echoed this advice, with one cautioning against allowing large draughts of cold air to chill the bodies of those sleeping and warning of the ‘evils arising from permanent dampness or humidity’ on board a ship.^87^ Ventilation was needed, but it must be with warm, dry air – a particularly hard commodity to come by in Antarctica. Balancing the need for warmth, ventilation and cleanliness became a source of much debate throughout the expedition. These debates expose how ideas about polar hygiene were contested.

Markham’s May 1900 report to the hygiene committee outlined the measures taken on previous polar expeditions to keep the air in the living quarters warm and dry.^88^ He was particularly taken with the idea of having a stove-warmed washing and bathing space in ‘communication with the open air, but not with the living deck’, which some nineteenth-century explorers had employed.^89^ This liminal space allowed the unsanitary and humid air produced by washing to escape from the ship, preventing it from causing damp in the living quarters. At the same time, the space provided a warmed area to wash in, protecting the vulnerable, naked bodies of the bathing men from the dangers of catching a chill. However, Markham notes that the schemes employed on these sailing ships ‘could not be adapted in a steamer’, as ‘the space could not be afforded’.^90^

Scott discusses the challenges of ventilating the ship in his published account. He notes that William Parry, the nineteenth-century Arctic explorer, claimed that ‘no artificial ventilation is necessary on a ship wintering in the Polar Regions, as the difference in temperature without and within is sufficient to cause a speedy interchange of air through the cracks or on the opening of doors’.^91^ However, Scott suggested that

Such a dictum might hold at a time when it was exceedingly difficult to make a ship airtight, and no doubt holds for our present condition on the *Discovery*; but if our decks had been thoroughly caulked some form of air inlet would have been necessary, and an ideal living-space for the polar regions should certainly possess a ventilating system capable of regulation and an entire freedom from casual draughts.^92^

Like Markham, Scott felt that the advances in maritime technology throughout the nineteenth century created additional challenges for preserving good health upon the ship, trapping the bodily emissions inside the ship and creating an extra need for artificial ventilation. Although the *Discovery* proved leaky, there can be little doubt that he was concerned by the issue of ventilation and saw the increasing ability of modern ships to trap air within them as a grave risk. For Scott, the threat came from too little contact between the explorers’ bodies and the fresh, clean, Antarctic air.

The concern with maintaining a warm, dry and well-ventilated ship occupied a significant proportion of the hygiene subcommittee’s three meetings.^93^ Information was gathered from Professor Erich von Drygalski on the German Antarctic expedition’s (1901–1903) plans for heating and ventilating their ship; although, Markham was sceptical about their proposals.^94^

**Figure 1.**
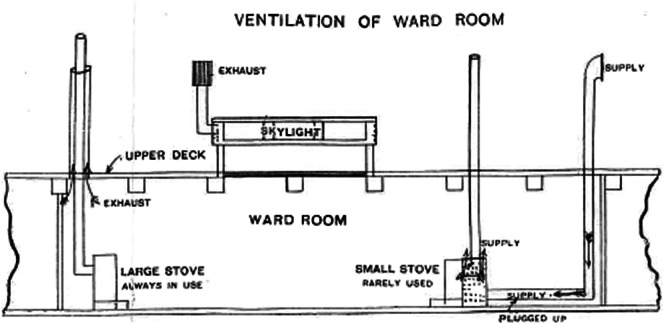
Ventilation System Employed on the Discovery. A similar system was employed in the crew’s living quarters. Scott, The Voyage of the Discovery, 1: 342.

Despite the interest the subcommittee took in this subject, there seems to have been little agreement on what action to take beyond the need for insulation and a general understanding that ventilation was important.^95^ The decisions on this matter were, in the end, taken by the ship’s designers and not the hygiene subcommittee.^96^ These resulted in a complex stove powered ventilation system being fitted to the ship (see Figure 1). The system employed involved fresh air being drawn into the ship through a warming chamber to ensure cold draughts did not enter the living quarters.^97^ Meanwhile, another stove’s exhaust draught was designed to ‘draw up’ the ‘vitiated air’ from the men’s living quarters, helping to ensure a healthy circulation of air into and out of the ship.^98^ In practice, Scott notes that ‘much of the theoretical benefit of this scheme vanished’, as ‘changes of wind’ produced ‘practical difficulties’, meaning ‘that there were times when the system was the object of universal contumely’.^99^ The benefits of the scheme were also reduced by the *Discovery*’s poor caulking, described above, which rendered the ship far less air tight than hoped; the system was also hampered by the large fuel consumption of two stoves. There thus seems to have been an over confidence in the ability of modern European technology to seal the men’s bodies off from the environment and the ability of technology to solve these problems.

The failure of the system meant that the ventilation of the officers’ wardroom was often dealt with by the far simpler method of opening or closing some skylights. But there was little consensus on how often this should happen. Consequently, ‘the community was divided into two camps, for and against the opening of skylights’, and as, initially, no agreement could be reached, ‘the skylights were continually flying up and down until a compromise was effected’. This settlement consisted of opening the doors and skylights in the morning ‘until the air was thoroughly renewed’ and then leaving them closed after this unless there was ‘general consent’ that the cabin had grown too hot or stuffy.^100^ Most agreed that cold draughts and ‘vitiated air’ had negative effects upon the body, but which of these two concerns should be the priority?

Scott notes that ‘the question of fresh air and ventilation was one that afforded us a constant field of argument’, writing that ‘even our medical officers were divided in opinion’.^101^ One of the doctors, Scott notes, continued to make ‘a bold stand for equable warmth, whilst the other contends that at all costs the purity of the air we breathed should be assured’.^102^ While Scott does not name the doctors in question, other incidents shed further light on the differing views of the expedition’s medical staff. Koettlitz, based on his own experiences on the Jackson–Harmsworth expedition, consistently argued that food rather than air or environment was the key factor in preserving health upon the ship.^103^ He had also been advised by Nansen, with whom he corresponded over a number of years, that ‘[v]entilation, warming etc. is very easily arranged and need[s] no special study’.^104^ Wilson, in contrast, often opened the wardroom skylights before breakfast. Scott notes there was ‘no complaint about the freshness of the air’ when he had done so ‘though occasionally people appear at the breakfast table in fur mitts as a mute protest against the temperature’.^105^ Wilson wrote at length about the hygiene and dampness of the ship in his report on the expedition, again illustrating his support for environmental explanations of disease.^106^

The different attitudes towards the relative importance of air were vividly illustrated in a debate between the expedition’s two medics over the causes of a scurvy outbreak, first detected in October 1902.^107^ Koettlitz was a firm supporter of the view that tainted meat was the cause of scurvy, even before the start of the outbreak.^108^ He was also sceptical of environmental explanations, noting that Nansen had avoided the illness by eating fresh meat despite being ‘in a far worse position and under worse conditions than any other polar expedition has ever been placed’.^109^ Based on these views, Koettlitz advised that the outbreak would ‘quickly disappear if fresh seal meat is the staple meat diet for every member of the expedition’.^110^ In contrast, Wilson suspected the environment of the ship as being a major cause of the outbreak. Writing in his diary at the time, he noted that the issue of damp on the ship had recently ‘become infinitely worse on account of the thaw’ and that ‘everything which was frozen and consequently dry, a short while back is now damp or wet or trickling’.^111^ Wilson then notes that this increase in dampness ‘coinciding with an outbreak of sickness is no unlucky accident’.^112^ The departure of the polar winter and the accompanying thaw – which transformed clean ice into potentially unhygienic and damp causing water – was, for Wilson, the probable cause of the scurvy outbreak.

Balancing these conflicting ideas, Scott adopted a dual track approach. On the one hand, efforts were made to clean the air on and disinfect the ship based on the understanding that ‘scurvy depends largely on environment, and there can be no doubt that severe or insanitary conditions of life contribute to the ravages of the disease’.^113^ Based on the view that dampness had caused, or at least contributed to, the scurvy problem, the bilges were pumped and the hold ‘cleaned, disinfected and whitewashed’ in order to remove the threat of miasmic gasses or biological contaminants escaping from this stagnant water.^114^ Meanwhile, the rest of the ship was scrubbed until everything was ‘as clean as a new pin’.^115^ The drying of clothes and sleeping bags on the mess deck was banned, the men’s bedding was aired, and other small measures introduced in an effort to improve both the warmth and dryness of the men’s living quarters.^116^ Scott also decided to give ‘everyone on the mess-deck a change of air’ and a small living space was established in the expedition’s hut for this purpose; exercise outdoors was also encouraged.^117^ On the other hand, following Koettlitz’s advice, efforts were also made to increase the supply and intake of fresh meat and parties were sent out to kill more seals – an activity that also provided an opportunity for outdoor exercise. The ration of fresh meat replaced tinned foods on the dinner table.^118^

The efforts to clean the ship soon dispelled the idea that unsanitary conditions had caused the outbreak. On the 13 October 1902, Wilson wrote that the outbreak of scurvy was ‘very unaccountable, for nothing radically wrong with the hygiene has been discovered in the examination of even suspected things’.^119^ Much of the suspected flooring was found to be in good condition and its removal halted.^120^ Wilson also reports that the bilges, which had recently thawed and which were suspected of harbouring dangerous, putrefying water ‘were not anything like as bad as I had expected them to be and were apparently less so than is commonly the case in a wooden ship’.^121^ Scott also notes that the inspections found all the bedding ‘quite dry’ and no significant build ups of dirt on the ship. ^122^ Consequently, Scott felt that the outbreak ‘could not possibly have come from insanitary conditions’.^123^ The results of this cleaning regime also led Wilson to change his views about the causes of the outbreak, and he notes that ‘[i]t seems almost necessary to fall back on the tinned food for explanation and yet these have been regularly and systematically examined’ and ‘[n]ot a tin of suspicious food ever passed’.^124^ The scurvy outbreak became a way to put competing ideas about the relative importance of air and food to the test, but the results were far from conclusive.^125^

One area where there was more consensus was the importance of outdoor exercise. Exercise played an increasingly important role in ideas about hygiene in the late nineteenth and early twentieth century. As Vanessa Heggie has argued, ‘mainstream medical opinion was in favour of vigorous exercise by 1900, at least for the “normal” person, that is the able-bodied adult male’.^126^ Within Anglo-American culture, physical exertion was seen as having both hygienic and educative effects, preventing disease but also bringing the body under the control of the mind through training the nervous system.^127^ Supporters viewed ‘proper exercise’ as ‘one way to minimize such physiological hazards of modern society as overcrowding, impure air, bad water, exhausting labor, and excessive “brain work.”’^128^ The importance of exercise was intertwined with the importance of fresh air, discussed above.^129^ Outdoor physical exertion was thus a means of preserving health whatever other risks the explorers were exposed to.

During the summer sledging seasons in Antarctica, back-breaking exercise was a central part of the explorers’ daily routine. In the winter, when the men spent most of their days on board *Discovery*, getting enough exercise was more complicated. Scott notes that, for the most part, there was no compulsion in the exercise routine during the polar winters. But he claims that ‘the men are intelligent enough to appreciate the advantage of good health and the benefit of a daily walk’. Special provisions were made so that those whose duties made walking difficult, such as the cook and stewards, had time for a ‘walk abroad’. ^130^ These provisions were increased during the scurvy outbreak to try and prevent the spread of the disease.^131^

The importance of exercise on the expedition reflected the growing promotion of games and sport within the Royal Navy in this period.^132^ Both officers and men played football when the weather allowed them to.^133^ In the expedition’s second winter in Antarctica, they played hockey, a game that became a source of much excitement but was also ‘capital exercise’.^134^ The preference for such games reflects the growing importance of team sports in British culture in this period, which sought to channel men’s energy into ‘controlled and acceptable competition’.^135^ But it also shows that enjoyment and play were important parts of the explorers’ fitness routines. Indeed, they began tobogganing down a slope near the ship, with many finding this a more enjoyable way to exercise than either walking or skiing.^136^ Some found walking in the same area quite boring. Armitage complained that during the winter ‘the daily exercise became so monotonous that I, for one, neglected it far more than I ought to have done, but cannot say that I felt any bad effects through doing so – although there is no doubt that one felt better after a sharp walk over the ice, especially if the weather was fine and there was no wind’.^137^

Exercise also allows us to see the psychological dimensions of polar hygiene. Scott notes the ‘curious fact’ that ‘throughout most of the winter most of the officers have preferred to take their daily walk alone’.^138^ Scott emphasised that the officers were ‘not at all sick of each other’s company’ and that the solitary nature of these walks was down to the difficulty of coordinating plans and communicating while dressed for the polar winter.^139^ While there may well be truth in these logistical considerations, his statement may reflect the need to demonstrate to a domestic audience a picture of good morale and fraternal masculine relations aboard the ship. Bruno Bouvel’s more recent study of groups of men overwintering in Antarctica found that individuals often expressed a desire for privacy and isolation driven by ‘a need to resist the anxieties of annihilation provoked by the large group’.^140^ While Edwardian views of privacy and individuality would have differed from contemporary ideas, Bouvel’s study suggests that there was a significant psychological dimension to the officers’ walks during the Antarctic winter. Exercise may have helped the officers to maintain a sense of individuality and identity in an environment where privacy and solitude were hard to come by.

Regimes of exercise also show the importance of working-class agency in the history of polar hygiene. Many of the sailors had been specifically selected for their strong physiques and had, therefore, already incorporated exercise into their daily routines. The original advert put out for volunteers to join the expedition claimed that, amongst other things, men would be ‘selected for their physique’.^141^ Many of the sailors had impressively muscular bodies. Scott commented ‘what a splendid set of men we have from the point of view of physique. Some turn the scale at over 190 lbs., and several at over 180 lbs., without an ounce of superfluous fat’.^142^ Armitage remarked that the crew’s ‘physical proportions would have called forth admiration from the Sandowists’.^143^ His reference here is to the followers of Eugen Sandow, the pioneer of modern bodybuilding, who gained popularity in the late nineteenth and early twentieth century and made a living by displaying his muscular body.^144^ Petty Officer Edgar Evans and stoker William Lashly both had particularly strong bodies. Scott described Evans as ‘a man of herculean strength’.^145^ Likewise, Scott claimed that Lashly was ‘never in anything but the hardest [physical] condition.^146^ These impressive bodies were often the result of many years of training. Evans was a physical training instructor within the Royal Navy before joining the expedition.^147^

Exercise was one of the few issues on which the expedition’s two medics agreed. Wilson’s medical report – under the heading ‘Hygiene of the Ship’ – notes that the men generally support this regime of outdoor exercise as it ‘enabled them to eat more heartily and to sleep; indeed the air and exercise were the secret of both’.^148^ Koettlitz also saw it as important. When commenting on the fitness of one of the men who had suffered from scurvy, he recommended a ‘a slight alteration in his duties which would give him more fresh air and exercise’, to help preserve his health if he stayed another winter.^149^ Outdoor exercise was a rare point of unanimity in the broader medical debates of the expedition.

## Conclusion

Discussions of hygiene on the BNAE mirror broader debates about preventative health care at the turn of the twentieth century. Like medics in other contexts, the organisers and explorers were worried about the quality of the food they ate, the purity of the air they breathed and encouraged regimes of exercise. Many of their concerns reflect broader anxieties about preserved food and bad air that were common across a variety of environmental settings. When it came to food, trust was also a key theme in these debates. By allowing food to be consumed ‘out of season and out of place’,^150^ tinned food removed explorers control over what they were putting into their bodies, and with dubious canning practices apparently prevalent, the consumption of tinned food provoked anxiety. This anxiety meant that the expedition was constantly working to establish which foods were safe to eat. Both in London and Antarctica, the safety of food was generally established not through scientific tests, but through personal experiences, embodied observations and sensory tests.

But there were also differences between polar hygiene and approaches in other environmental settings. In the tropics, proponents of hygiene theory generally sought to create a barrier between the explorer and the environment. On Antarctic expeditions, the relationship between hygiene and the environment was viewed in different terms. Here, it was the explorers’ bodies, ship and food supplies that were viewed as some of the main threats. The outside environment was not seen as a potentially contaminating space by those on the expedition but was, instead, viewed as a place where clean air and nontainted food could be obtained. But environment did play an important role in the testing of food. The tropics were seen as the main and most rigorous test of tinned food’s quality, rendering visible to the doctors’ senses any underlying but otherwise imperceptible imperfections.

The outbreak of scurvy became a way to test ideas about the relative importance of different threats to the human body. However, it also exposed the limits of medical knowledge, as while food was identified as the source of the outbreak, there was little agreement on what exactly was wrong with the food and no conclusive evidence that would settle the debate. In this sense, the BNAE must also be understood within a longer tradition of British naval medicine, which had sought to use hygienic measures to prevent outbreaks of scurvy on polar expeditions. The organisers of the BNAE drew on the experience of an array of medics, scientists and explorers, seeking to use their expertise to systematically study the problem of polar health. The ultimate failure of these efforts to secure wholesome food, clean air and avoid scurvy outbreaks illustrated the limits of these methods. The later expeditions of Scott and Shackleton still sought to prevent the disease through adopting hygienic precautions (with varying degrees of success), but compared to the BNAE their efforts were comparatively ad hoc.

The expedition employed a variety of tests to establish if the food they ate was safe, experimenting with both scientific tests and examinations at victualing yards. In practice, though, the explorers fell back on the medics’ sensory assessments of the relative safety of different food items and physical examinations of the men’s living quarters. As such, the expedition tells us something more general about the importance of trust and sensory labour within medical practice in this period. Medical work in this context involved having disciplined and attentive senses, hopefully able to detect signs of damp bedding, stale air or decomposition and decay in food supplies. In this sense, there are clear parallels between the activities of these polar medics and more recent literature that has sought to understand medicine as a ‘craft’.^151^

## Footnotes

1

Vanessa Heggie , ‘Blood, Race and Indigenous Peoples in Twentieth Century Extreme Physiology’, History and Philosophy of the Life Sciences, 41, 2 (June 13, 2019), 26 31197607 10.1007/s40656-019-0264-zPMC6565645; Vanessa Heggie , Higher and Colder: A History of Extreme Physiology and Exploration (University of Chicago Press, 2019); Vanessa Heggie, ‘Why Isn’t Exploration a Science?’, *Isis*, 105, 2 (2014), 318–34.

2

Jason C. Anthony , Hoosh: Roast Penguin, Scurvy Day, and Other Stories of Antarctic Cuisine (Lincoln and London: University of Nebraska Press, 2012); Edward Armston-Sheret , ‘“A Good Advertisement for Teetotalers”: Polar Explorers and Debates over the Health Effects of Alcohol, 1875–1904’, The Social History of Alcohol and Drugs, 33, 2 (August 28, 2019), 257–85; Robert Earl Feeney , Polar Journeys: The Role of Food and Nutrition in Early Exploration (Fairbanks: University of Alaska Press, 1997 ); Henry R. Guly, ‘Medical Comforts during the Heroic Age of Antarctic Exploration’, *The Polar Record*, 49, 2 (April 2013), 110–17; Henry Guly, ‘The Understanding of Scurvy during the Heroic Age of Antarctic Exploration’, *Polar Record*, 49, 1 (January 2013), 26–32; Bernadette Hince , ‘‘Seal Liver and Onion for Dinner’: The Role of Food in Preserving the Peace on Some Early Antarctic Expeditions’, The Polar Journal, 4, 2 (July 3, 2014), 377–88.

3 For the broader history of the expedition, see T. H. Baughman , Pilgrims on the Ice: Robert Falcon Scott’s First Antarctic Expedition (Lincoln, Neb.: Nebraska University Press, 2000); David Yelverton , Antarctica Unveiled: Scott’s First Expedition and the Quest for the Unknown Continent (Denver: Colorado University Press, 2000).

4 See, in particular, Antarctic Expedition Collection [hereafter NAE Collection], AA, Royal Geographical Society (with IBG) archives [hereafter RGS].

5 The subcommittee met three times, in May and July 1900, and, after a long break, in March 1901 the time of the last meeting, Scott replaced Lord Lister as the committee’s chair. Amongst the rest of the subcommittee’s membership, the medical profession was well represented: four of the five other permanent members were doctors. The Navy was represented by Vice Admiral Albert Markham.

6 Clements Markham letter to Robert F. Scott, 26 June 1899, Scott Polar Research Institute Archive [hereafter SPRI], MS366/15/26/2, University of Cambridge.

7 Copy of Robert F. Scott letter to Presidents of the Royal Society and Royal Geographical Society, undated, SPRI, MS 366/11/60;ER, University of Cambridge.

8 See Reginald Koettlitz letter to Robert F. Scott, 9 November 1900, NAE Collection, AA/7/1/2, RGS Archives.

9

George Wilson , A Handbook of Hygiene (J & A Churchill, London, 1873), 7–8.

10 Michael Worboys, *Spreading Germs: Disease Theories and Medical Practice in Britain, 1865–1900* (Cambridge: Cambridge University Press, 2000); Johannes Fabian, *Out of Our Minds: Reason and Madness in the Exploration of Central Africa* (Berkley: University of California Press, 2000), 61; Laura Newman, *Germs in the English Workplace, c.1880–1945* (London: Routledge, 2021). On the persistence of miasmic theories, see Stephen Halliday, ‘Death and Miasma in Victorian London: An Obstinate Belief,’ *BMJ*, 323, 7327 (December, 2001), 1469–71. On representations of miasmas, see Amanda Sciampacone,’ “Epidemics in a Mist”: Medical Climatology and Cholera in Victorian Visual Culture’, *Journal of Victorian Culture*, 25, 4 (November, 2020): 492–511.

11 John Snow, *One the Mode of Communication of Cholera,* (London, 1855); Joseph Lister , ‘On the Antiseptic Principle in the Practice of Surgery’, BMJ, 2, 351 (1867), 246–4810.1136/bmj.2.351.246PMC231061420744875; Nancy Tomes , ‘The Private Side of Public Health: Sanitary Science, Domestic Hygiene and the Germ Theory, 1870–1900’, Bulletin of the History of Medicine, 64, 4 (1990), 509–392073544 . On commodities, see Ryan Johnson, ‘European Cloth and ‘Tropical’ Skin: Clothing Material and British Ideas of Health and Hygiene in Tropical Climates’, *Bulletin of the History of Medicine*, 2009, 530–60; Ryan Johnson , ‘Tabloid Brand Medicine Chests: Selling Health and Hygiene for the British Tropical Colonies’, Science as Culture, 17, 3 (September 1, 2008), 249–68. For another setting see, Catherine Mills , ‘The Emergence of Statutory Hygiene Precautions in the British Mining Industries, 1890–1914’, The Historical Journal, 51, 1 (March 2008), 145–68.

12

Eliza Juzda Smith , ‘Cleanse of Die: British naval Hygiene in the Age of Steam, 1840–1900’, Medical History, 66, 2 (2018), 177–9810.1017/mdh.2018.3PMC588316429553010; See also, Paul E. Sampson, ‘“The Lungs of a Ship”: Ventilation, Acclimatization, and Labor in the Maritime Environment, 1740–1800’, *History of Science*, 61, 2 (June 1, 2023), 214–35. On merchant seaman’s health in this period, see Tim Carter, *Merchant Seamen’s Health, 1860–1960: Medicine, Technology, Shipowners and the State in Britain* (Boydell & Brewer Ltd., 2014). The BNAE was, however, organised largely on naval lines.

13 Smith, *op. cit.* (note 12), 178–9.

14 Smith, *op. cit.* (note 12), 178–79.

15 Smith, *op. cit.* (note 12), 181.

16 Smith, *op. cit.* (note 12), 191.

17 Akwasi Kwarteng Amoako-Gyampah, ‘Household Sanitary Inspection, Mosquito Control and Domestic Hygiene in the Gold Coast [Ghana] from the Late-Nineteenth to the Mid-Twentieth Century’, *Social History of Medicine*, 35, 1 (February 1, 2022), 278–301.

18 Fabian, *op cit.* (note 10), 61–2; On this issue more broadly see, Mark Harrison , Climates and Constitutions: Health, Race, Environment, and British Imperialism in India, 1600–1850 (Oxford: Oxford University Press, 1999). See also, Alison Bashford , Imperial Hygiene: A Critical History of Colonialism, Nationalism and Public Health (Basingstoke: Palgrave Macmillan, 2004).

19

David N. Livingstone , ‘Tropical Climate and Moral Hygiene: The Anatomy of a Victorian Debate’, The British Society for the History of Science, 32 (1991), 93–110 10.1017/s000708749800350111623678.

20 Livingstone, *ibid.*

21 Livingstone, *ibid*, 94.

22

Edward Armston-Sheret and Kim Walker , ‘Is Alcohol a Tropical Medicine? Scientific Understandings of Climate, Stimulants and Bodies in Victorian and Edwardian Tropical Travel’, The British Journal for the History of Science, 54, 4 (2021), 465–8434558394 10.1017/S0007087421000649.

23 On the development of tropical medicine see Michael Worboys , ‘Germs, Malaria, and the Invention of Mansion Tropical Medicine: From “Diseases in the Tropics” to ‘Tropical Diseases’, in David Arnold (ed.), Warm Climates and Western Medicine: The Emergence of Tropical Medicine 1500–1900, (Leiden: Brill, 1996), 181–207.8783626

24

Jonathan Lamb , Scurvy: The Disease of Discovery (Princeton: Princeton University Press, 2016), 12.

25 Thomas Trotter, *Observations on the Scurvy* (Edinburgh: Charles Elliot, and G.G. J. and J. Robinson, 1786), 28–9.

26 Trotter, *ibid.*, 28–9.

27

Kenneth Carpenter , The History of Scurvy and Vitamin C (Cambridge: Cambridge University Press, 1986), 51–74.

28 Carpenter, *ibid.*, 96.

29 Carpenter, *ibid.*, 118, 129; Edward Armston-Sheret , ‘Tainted Bodies: Scurvy, Bad Food and the Reputation of the British National Antarctic Expedition, 1901–1904,’ Journal of Historical Geography, 65 (July, 2019), 19–28 .

30 Carpenter, *ibid.*, 116, 119.

31

Robert F. Scott , The Voyage of the ‘Discovery’, 2 vols. (London: Smith and Elder, 1905), 1: 530 ; see also Guly, ‘The Understanding of Scurvy’, *op cit.* (note 2).

32

Alexander Armstrong , Observations on Naval Hygiene and Scurvy: More Particularly as the Latter Appeared during a Polar Voyage (London: John Churchill, 1858), 32–3, 86 PMC518325430163927.

33 Armstrong, *ibid.*, 33–4.

34 Fridtjof Nansen letter to Reginald Koettlitz 27 Feb 1900, Reginald Koettlitz Collection, LMS K12, RGS.

35 Clements Markham, Report to Hygiene Sub-Committee, 7 May 1900, NAE Collection, AA/6/2/2/4, RGS.

36 Markham, *ibid.*, f. III.

37 Markham, *ibid.*, f. IV.

38 Markham, *ibid.*, f. II

39 Markham, *ibid.*, f. II.

40 Markham, *ibid.*, f. IV.

41 Reginald Koettlitz letter to Robert F. Scott, 9 November 1900, NAE Collection, AA/7/1/2, RGS.

42 Koettlitz, *ibid.*

43 Koettlitz, *ibid.*

44 Scott, *op. cit.* (note 31), 1: 441.

45 Reginald Koettlitz letter to Robert F. Scott, 15 November 1900, NAE Collection, AA/7/1/3 Collection, RGS.

46

Shane McCorristine and Jane S. P. Mocellin , ‘Christmas at the Poles: Emotions, Food, and Festivities on Polar Expeditions, 1818–1912’, Polar Record, 52, 5 (2016), 562–77.

47 On this point, see Boucher, Ellen . ‘Arctic Mysteries and Imperial Ambitions: The Hunt for Sir John Franklin and the Victorian Culture of Survival’, Journal of Modern History, 90, 1 (2018), 49–75 .

48 Clements Markham to Reginald Koettlitz, 29 October 1900, Reginald Koettlitz Collection, LMS K12, RGS. On broader attitudes to preserved meat in this period, see Berris Charnley , ‘Arguing over Adulteration: The Success of the Analytical Sanitary Commission’, Endeavour, 32, 4 (December 1, 2008), 129–3319042021 10.1016/j.endeavour.2008.10.003; Rebecca J. H. Woods , ‘The Shape of Meat: Preserving Animal Flesh in Victorian Britain’, Osiris, 35 (August 2020), 123–41.

49

Frederick G. Jackson , and Vaughan Harley ‘An Experimental Inquiry into Scurvy’, Proceedings of the Royal Society of London, 66 (1899), 250–65.

50

Anne Hardy , ‘Food, Hygiene, and the Laboratory. A Short History of Food Poisoning in Britain, circa 1850–1950’, Social History of Medicine, 12, 2 (August 1, 1999), 293–311 11623930 10.1093/shm/12.2.293.

51 Clements Markham, Memo on Provisions Recommended by Nansen, undated, NAE Collection, AA/6/2/5, RGS.

52 Markham, *op cit.* (note 35), f. IV.

53 Markham, *op cit.* (note 35), f. IV.

54 Simon Naylor, ‘Spacing the Can: Empire, Modernity, and the Globalisation of Food’, *Environment and Planning A: Economy and Space,* 32, 9 (1 September 2000), 1625–39.

55 Dr H. Armstrong, Health Department, Newcastle on Tyne, to Clements Markham, 8 June 1900, NAE Collection, AA/6/2/8, RGS.

56 Food List for 46 Men for Three Years, undated, NAE Collection, AA/6/2/6, RGS.

57 T. E. Thorpe letter to Cyril Longhurst, NAE Collection, 26 February 1901, AA/6/2/10, RGS.

58 Thorpe, *ibid.*

59 T. E. Thorpe letter to Cyril Longhurst, NAE Collection, 26 February 1901 to 6 June 1901, AA/6/2, RGS.

60 T. E. Thorpe letter to C. Longhurst, NAE Collection, May 1901, AA/6/2/10, RGS.

61 Thorpe, *op. cit.* (note 59).

62 Thorpe, *op. cit.* (note 59).

63 Clements Markham letter to Horace Walpole, 23 April 1903, NAE Collection, AA/6/4/3, RGS.

64 Markham, *ibid.*

65 H. Spadaccini letter to Robert F. Scott, 15 June 1901, NAE Collection, AA/6/3/4, RGS.

66 Markham, *op cit.* (note 63).

67 Markham, *op cit.* (note 63).

68 H. Spadaccini letter to Robert F. Scott, 19 June 1901, NAE Collection, AA/6/3/11, RGS.

69 H. Spadaccini letter to Robert F. Scott, 17 July 1901, NAE Collection, AA/6/3/9, RGS.

70 H. Spadaccini letter to Robert F. Scott, 19 July 1901, NAE Collection, AA/6/3/11, RGS

71 Reginald Koettlitz letter to Robert F. Scott, 6 October 1902, SPRI, MS 366/14/27;ER, University of Cambridge.

72 Robert F. Scott’s annotation on A.B. Kempe Report Memorandum as to Provisions, 11 March 1905, SPRI, MS366/14/28;ER, University of Cambridge.

73 Scott, *op. cit.* (note 31), 1: 307.

74

Edward Wilson , Diary of the ‘Discovery’ Expedition to the Antarctic Regions 1901–1904, edited from the original manuscript by Ann Savours (London, Blandford Press, 1966), 151.

75 Wilson, *ibid.*, 151.

76 Scott, *op. cit.* (note 31), 1: 336.

77 Scott, *op. cit.* (note 31), 1: 100.

78

Albert Armitage , Two Years in the Antarctic: Being a Narrative of the British National Antarctic Expedition (London: Edward Arnold, 1905), 138–42.

79 Armitage, *ibid.*, 88.

80 Markham, *op. cit.* (note 35), RGS, f. V.

81 Scott, *op. cit.* (note 31), 1: 342.

82

John Macdonald , Questions of Naval Hygiene (London: Smith and Elder, 1881), 4.

83

Peter Baldwin , ‘How Night Air Became Good, 1776–1930’, Environmental History, 8, 3 (2003), 415 .

84 Macdonald, *op cit.* (note 82), 90.

85 Sampson, *op cit.* (note 12), 235.

86 Baldwin, *op cit.* (note 83), 415

87 Macdonald, *op cit.* (note 82), 53: 162.

88 Markham, *op. cit.* (note 35), RGS.

89 Markham, *op. cit.* (note 35), V; On heating arrangements on other expeditions, see Admiralty. Committee on Scurvy, *Report of the Committee Appointed by the Lords Commissioners of the Admiralty, to Enquire into the Causes of the Outbreak of Scurvy in the Recent Arctic Expedition; the Adequacy of the Provision Made by the Admiralty in the Way of Food, Medicine, and Medical Comforts ; and the Propriety of the Orders Given by the Commander of the Expedition for Provisioning the Sledge Parties* (London: Government Stationery Office, 1877), minutes of evidence, page 14.

90 Markham, *op. cit.* (note 35), VI.

91 Scott, *op. cit.* (note 31), 1: 341.

92 Scott, *op. cit.* (note 31), 1: 341–42.

93 Anon., *Minute Book of the National Antarctic Expedition*, NAE Collection, AA/20/29, RGS.

94 Anon., *ibid.,* entry 10 July 1900. See also Markham, *op. cit.* (note 35), f. VI.

95 Anon., *op. cit.* (note 93), entry 10 July 1900; Markham, *op. cit.* (note 35).

96 For a summary of the different committee meetings see Anon., *op. cit.* (note 93). Papers of the ship committee can be found in files NAE collection files AA5/2/1–39, RGS see in particular W. E. Smith, ‘On the Design of the Antarctic Exploration Vessel “Discovery”’ as read at the spring meetings of the Forty-Sixth Session of the Institution of Naval Architects, 12 April 1905, NAE collection, AA/5/2/39, RGS.

97 Scott, *op. cit.* (note 31), 1: 343.

98 Scott, *op. cit.* (note 31), 1: 343.

99 Scott, *op. cit.* (note 31), 1: 343.

100 Scott, *op. cit.* (note 31), 1: 343.

101 Scott, *op. cit.* (note 31), 1: 343.

102 Scott, *op. cit.* (note 31), 1: 343–4.

103 Reginald Koettlitz, ‘National Antarctic Expedition’, *BMJ*, 1, 2145 (February 1902), 342–3. See also Jackson and Harley, *op. cit.* (note 49), 250 –265, which summarises the Jackson–Harmsworth expedition’s argument that tainted food is the causes of scurvy.

104 Nansen *op. cit.* (note 34).

105 Scott, *op. cit.* (note 31), 1: 217.

106 Edward Wilson ‘The Medical Aspect of the *Discovery’s* Voyage to the Antarctic’, *BMJ*, 2, 2323 (July 1905), 79.

107 Reginald Koettlitz letter to Robert F. Scott, 3 October 1902, SPRI, MS 366/14/23;ER, University of Cambridge.

108 Koettlitz, *op. cit.* (note 103), 343; Clements Markham letter to Horace Walpole, 27 May 1903, NAE Collection, AA/6/4/5, RGS

109 Koettlitz, *op. cit.* (note 103), 342.

110 Koettlitz, *op. cit.* (note 107).

111 Wilson, *op. cit.* (note 74), entry Sunday 5 October 1902, 196–7.

112 Wilson, *op. cit.* (note 74), entry Sunday 5 October 1902, 196–7.

113 Scott, *op. cit.* (note 31), 1: 555.

114 Wilson, *op. cit.* (note 74), entry Sunday 5 October 1902, 196–7.

115 Scott, *op. cit.* (note 31), 1: 543; See also, Armitage, *op. cit.* (note 78), 138.

116 Wilson, Op Cit. (note 74), entry Sunday 5 October 1902, 196–7.

117 Scott, *op. cit.* (note 31), 1: 543, 545; Armitage, *op. cit.* (note 78), 138.

118 Scott, *op. cit.* (note 31), 1: 544.

119 Wilson, *op. cit.* (note 74), Monday 13 October 1902, 201.

120 Wilson, *op. cit.* (note 74), Monday 13 October 1902, 197.

121 Wilson, *op. cit.* (note 74), Monday 13 October 1902, 201.

122 Scott, *op. cit.* (note 31), 1: 543.

123 Scott, *op. cit.* (note 31), 543

124 Wilson, *op. cit.* (note 74), Monday 13 October 1902, 201.

125 Wilson, *op. cit.* (note 74), Monday 13 October 1902, 201.

126 Vanessa Heggie, ‘Bodies, Sport and Science in the Nineteenth Century,’ *Past & Present*, 231, 1 (May 1, 2016), 180. More broadly, see Bruce Haley , The Healthy Body and Victorian Culture (Cambridge, Mass.: Harvard University Press, 1978).

127 Roberta J. Park, ‘Physiologists, Physicians, and Physical Educators: Nineteenth Century Biology and Exercise, “Hygienic’ and ‘Educative”’, *Journal of Sport History*, 14, 1 (1987), 28–60. Ideas about the importance of exercise were not monolithic. One strand of thinking were ideas of ‘muscular Christianity’, which emphasised ‘physical strength, religious certainty, and the ability to shape and control the world around oneself’. Donald E. Hall , ‘Introduction: Reading and Writing the Male Social Body’, in Donald E. Hall (ed.), Muscular Christianity: Embodying the Victorian Age (Cambridge: Cambridge University Press: 1994), 7 . For a summary of different schools of thought on this subject, see Heggie, *op. cit.* (note 126), 172–80.

128 Park, *op. cit.* (note 127), 42.

129 In contrast, travellers in the tropics often complained about the quality of the air they breathed. Edward Armston-Sheret , ‘Recentring the Body in Histories of British Exploration, c.1850–1914’ (PhD thesis, Royal Holloway University of London, 2021), 286–9.

130 Scott, *op. cit.* (note 31), 1: 296.

131 Armitage, *op. cit.* (note 78), 138.

132

Tony Mason and Eliza Riedi , Sport and the Military: The British Armed Forces 1880–1960 (Cambridge: Cambridge University Press, 2010), 15–49.

133 Scott, *op. cit.* (note 31), 1: 305, 545.

134 Scott, *op. cit.* (note 31), 2: 182.

135 Heggie, *op. cit.*(note 126), 175.

136 Scott, *op. cit.* (note 31), 1: 308.

137 Armitage, *op. cit.* (note 78), 216.

138 Scott, *op. cit.* (note 31), 1: 400.

139 Scott, *op. cit.* (note 31), 1: 400.

140 Bruno Bouvel, ‘No Exit’in Antarctica’, *Group Analysis*, 32, 3 (1999), 370.

141 Anon., ‘Service in the Antarctic Expedition’, 1901, NAE collection, RGS/AA/4/1, RGS Archives, f. 1.

142 Scott, *op. cit.* (note 31), 1: 308.

143 Armitage, *op. cit.* (note 78), 121.

144 On Shackleton’s 1907–09 expedition some explorers did ‘Sandow exercises daily’. Ernest Shackleton , The Heart of the Antarctic (London: Heinemann 1909), 1: 144 .

145 Scott, *op cit.* (note 31), 2: 545.

146 Scott, *op cit.* (note 31), 2: 545.

147 See, Isobel Williams , Captain Scott’s Invaluable Assistant: Edgar Evans (Cheltenham: The History Press, 2012).

148 Wilson, *op cit.* (note 106), 79.

149 Reginald Koettlitz letter to Robert F. Scott 19 February 1903, SPRI, MS 366/14/23;ER, University of Cambridge.

150 Naylor, *op cit.* (note 54), 1633.

151 E.g., Simon Carmel , “The Craft of Intensive Care Medicine,” Sociology of Health & Illness, 35, 5 (2013): 731–4523009749 10.1111/j.1467-9566.2012.01524.x.

