# Nourishing food, clean air and exercise: medical debates over environment and polar hygiene on Robert Falcon Scott’s British National Antarctic expedition, 1901–1904 – CORRIGENDUM

**DOI:** 10.1017/mdh.2025.10033

**Published:** 2026-01

**Authors:** Edward Armston-Sheret

**Keywords:** Hygiene, exploration, Edwardian, polar, Antarctic, scurvy, corrigendum

The author apologises for an error in the published article. On the second page of the article, lines 7-8 contain an error. The sentence currently reads: “This vessel was manned by a slightly varying crew of just under forty officers, scientists and sailors.”

But it should read: “This vessel was manned by a slightly varying crew of just under fifty officers, scientists and sailors.”
